# Effect of Population Lockdown on Pediatric Emergency Room Demands in the Era of COVID-19

**DOI:** 10.3389/fped.2020.00521

**Published:** 2020-09-18

**Authors:** Francesco Valitutti, Letizia Zenzeri, Angela Mauro, Rosario Pacifico, Micaela Borrelli, Stefania Muzzica, Giovanni Boccia, Vincenzo Tipo, Pietro Vajro

**Affiliations:** ^1^Pediatric Unit, AOU San Giovanni di Dio e Ruggi D'Aragona, Salerno, Italy; ^2^EBRIS (European Biomedical Research Institute of Salerno), Salerno, Italy; ^3^Emergency Pediatric Department, AORN Santobono-Pausilipon Children's Hospital, Naples, Italy; ^4^Pediatric Surgery Unit, AOU San Giovanni di Dio e Ruggi D'Aragona, Salerno, Italy; ^5^Hospital Hygiene and Epidemiology Unit, Department of Medicine, Surgery and Dentistry, University of Salerno, Baronissi, Italy; ^6^Clinical Pediatrics Unit, Department of Medicine, Surgery and Dentistry, University of Salerno, Baronissi, Italy

**Keywords:** COVID-19, lockdown, emergency department, overcrowding, appropriateness, children

## Abstract

**Objectives:** The aim of this study was to assess the impact of the COVID-19 pandemic and population lockdown on pediatric ED consultations.

**Methods:** A cross-sectional study on pediatric emergency department consultations before and during the current COVID-19 pandemic (March–May 2019 vs. March–May 2020) was performed in two hospitals in the Campania region (Southern Italy) [i.e., Salerno University Hospital (Salerno) and Pediatric Regional Referral Emergency Hub “AORN Santobono-Pausillipon” (Naples)].

**Results:** 29,368 consecutive ED pediatric patients (13,430 females; mean age ± *SD* = 5.4 ± 4.7 years) were seen in March–May 2019 and 9,133 (4,494 females; mean age ± *SD* = 5.9 ± 4.2 years) in March–May 2020. Resuscitation/emergency and urgent care pediatric ED consultations were 1,388 (4.7%, 95% CI 4.5–4.9) in the 2019 trimester, while they were 648 (7.1%, 95% CI 6.6–7.6) in the 2020 trimester (*p* < 0.01). Mean pediatric ED daily consultations were 326.3 (95% CI 299.9–352.7) in the considered period of 2019 and 101.4 (95%CI 77.9–124.9) in the same period of 2020 (*p* < 0.001). COVID-19 nasal swabs were performed for 385 children; of those, six resulted positive and four of them were hospitalized.

**Conclusions:** This work provides a unique snapshot of the pediatric EDs demands in the era of COVID-19. We witnessed a significant reduction of non-urgent health care demands during the pandemic but an increase of more severe urgent cases. The COVID-19 pandemic and the following lockdown unveiled the inappropriateness of the majority of pediatric ED consultations. Nevertheless, the current scenario highlighted the need for appropriate and timely clinical evaluations in the pediatric primary care to tackle late and more severe diagnoses in EDs.

## Introduction

Emergency department (ED) patient flow includes roughly 20–25% of pediatric consultations in western countries each day (1). In Italy, ED visits for children are quite often inappropriate, especially where pediatric primary care is well-structured and could be readily available (2). Since ED overcrowding is a multifaceted issue and a public health hazard, it requires committed policy and medical interventions in order to minimize its related risks (3). In fact, overcrowding in ED has been associated with poor hospital outcomes both in adults and children (4, 5); moreover, it is a well-recognized risk factor for infectious disease spreading (6). Interdisciplinary collaborative research and education are therefore still needed to develop and implement new solutions and strategies to both prevent and manage this serious drawback (7).

On February 21, 2020, the first case of coronavirus disease 2019 (COVID-19), also known as severe acute respiratory syndrome coronavirus 2 (SARS-CoV-2,) was diagnosed in Northern Italy (8), and a few weeks later, on March 11, 2020, the WHO declared officially it as a pandemic (9).

COVID-19 affects mainly adults whose clinical features display severe interstitial hypoxemic pneumonia (10), but children can be infected, spread the virus, and in almost all cases present a much milder disease (11, 12).

At the beginning of the COVID-19 epidemic, hospital-acquired coronavirus infection may have unknowingly fueled the rising epidemic curve (13), while in the following weeks the population lockdown has strongly tackled its spreading and was confirmed as an effective public health intervention (14).

The Italian government formally announced a national lockdown on March 9: this act implied the complete shutdown for schools, universities, public places, and all the shops but supermarkets, grocers, and pharmacies, along with well-defined traveling restrictions.

However, the current epidemic and population lockdown have dramatically changed worldwide the daily scenario in EDs as regards flows, paths, and disease categories of patients in pursuit of health care.

The aim of our cross-sectional study was to highlight the impact of the COVID-19 pandemic on ED consultation appropriateness for children in two hospitals of Southern Italy, comparing retrospective data from the trimesters March–May 2019 and March–May 2020.

## Materials and Methods

We retrospectively evaluated our pediatric ED files of two hospitals in the Campania Region (Southern Italy) [i.e., University Hospital “San Giovanni di Dio e Ruggi D'Aragona” (Salerno) and Pediatric Regional Referral Emergency Hub “AORN Santobono-Pausillipon” (Naples)]. Local internal review boards (Approved regional IRB protocol N. 10 on 28/04/2020 meeting) approved the retrospective chart review of electronic anonymized patient data. We compared medical files of trimester March–May 2019 (no COVID-19 pandemic) vs. files of trimester March–May 2020 (during COVID-19).

ED consultations were triaged by specialized nurses according to the Emergency Severity Index version 4 (ESI v.4), which included five items: (1) resuscitation, (2) emergency care, (3) urgent care, (4) less urgent care, and (5) non-urgent care (15). Appropriate ED visits were defined as: (1) ESI v.4 resuscitation, (2) ESI v.4 emergency care, (3) ESI v.4 urgent care, (4) ESI v.4 less urgent care that nevertheless required prompt investigations not available within the primary care, and (5) ESI v.4 non-urgent care that nevertheless required prompt investigations not available within the primary care.

Involvement of emergency medical transport services (EMTS) (i.e., ambulances) was taken into account and considered as another suitable approximation of clinical severity. EMTS appropriateness was defined as the ratio of urgent and emergency EMTS cases over total EMTS cases. Moreover, we looked at possible temporal patterns of ED consultations, primary care referral rates to the ED, numbers of inpatient hospital admissions, and mean inpatient hospital stay during the two considered periods.

Statistical analysis was performed using IBM SPSS Statistics (IBM Corp., Released 2010; IBM SPSS Statistics for Windows, NY, USA). Data are presented as absolute numbers, percentage, means, and standard deviations. Student's *t* or chi-squared tests were used to compare subgroup data as appropriate. A *p* < 0.05 was considered significant for all the tests performed.

## Results

Patient demographics and triage classification of the two groups are shown in Table 1.

**Table 1:** Patients demographics in the two considered periods.

	**March–May 2019**	**March–May 2020**
*n* of patients	29,368	9,133
Mean age in years (± standard deviation)	5.4 (± 4.7)	5.9 (± 4.2)
Females / Males ratio	13,430 / 15,938	4,494/4,639
Emergency consultations [i.e., ESI 1 and ESI 2](%)	34 (0.11%)	46 (0.50%)
Urgent consultations [i.e., ESI 3](%)	1,354 (4.61%)	602 (6.59%)
Non urgent consultations [i.e., ESI 4 and ESI 5](%)	27,980 (95.25%)	8,485 (92.88%)
EMTS involvement (%)	829 (2.82%)	381 (4.17%)
Inpatient hospital admissions from ED (%)	2,812 (9.57%)	1,455 (15.93%)

*ESI, emergency severity index; EMTS, emergency medical transports services; ED, Emergency Department*.

Study population included 38,501 children aged from 1 month to 14 years (mean age ± *SD*: 5.6 ± 4.5 years; 17,924 females) who accessed the EDs of the two hospitals during the two considered periods. Pediatric ED consultations were 29,368 in March–May 2019 and 9,133 in March–May 2020, which accounted for 68.9% reduction in ED utilization for children.

Resuscitation/emergency and urgent care pediatric consultations were 1,388 (4.7%, 95% CI 4.5–4.9) in the 2019 trimester, while they were 648 (7.1%, 95% CI 6.6–7.6) in the 2020 trimester (*p* < 0.01). Considering only most severe cases, resuscitation/emergency care ED demands for children were 34 (0.11%, 95% CI 0.08–0.14) in 2019 considered period and 46 (0.50%, 95%CI 0.36–0.64) in the same period of 2020 (*p* < 0.01). Of these, six patients in March–May 2019 and seven patients in March–May 2020 required ICU admission for mechanical ventilation (none of these were COVID-19 positive).

Appropriate consultations were 6,372 (21.7%, 95% CI 21.3–22.1) in 2019 trimester and 2,903 (31.8%, 95% CI 30.9–32.7) in 2020 trimester (*p* < 0.01).

Primary care referral rates to the EDs was 1.6% (95% CI 1.5–1.7) and 4.6% (95% CI 4.2–5.0) in the 2019 and 2020 trimesters, respectively (*p* < 0.01).

EMTS was involved for 829 children in March–May 2019 and for 381 children in March–May 2020. EMTS appropriateness was lower in the 3 months of 2019 rather than those of 2020 (12.3 and 18.3%, respectively; *p* < 0.01).

During night shifts (8 p.m.−8 a.m.), 98.9% (95% CI 98.6–99.2) of the health care requests accounted for non-urgent consultations in March–May 2019, while in March–May 2020 non-urgent consultations were 74.2% (95% CI 72.8–75.6) (*p* < 0.001).

During weekends (Saturdays and Sundays), 99.1% (95% CI 98.8–99.4) of the ED consultations were represented by non-urgent cases in the 2019 trimester, while in March–May 2020 they were 94.3% (95% CI 92.9–95.7) (*p* < 0.001).

Mean pediatric ED daily consultations were 326.3 (95% CI 299.9–352.7) in March–May 2019 and 101.4 (95% CI 77.9–124.9) in March–May 2020 (*p* < 0.001). Considering that the lockdown officially started on March 9, 2020, we saw higher daily consultations in the week from March 1 to March 8 2020 before lockdown (mean: 159.2; 95% CI 136.4–182.0) as compared with the after-lockdown time frame within the same month [i.e., from March 9 to March 31 2020 (mean: 63.3; 95% CI 56.9–69.7) (*p* < 0.001)].

Mean waiting time for non-urgent consultation was 55.2 min (*SD*: ± 45.2) in 2019 trimester and 23.1 min (*SD*: ± 15.7) in March–May 2020 (*p* < 0.001).

Table 2 summarizes the groups of signs and symptoms prompting ED consultations.

**Table 2:** Main health problems prompting emergency department consultations.

	**March–May 2019**	**March–May 2020**	
Fever	4,212 (14.3%)	1,477 (16.2%)	*p* < 0.001
Respiratory distress	2,792 (9.5%)	1,013 (11.1%)	*p* < 0.001
Trauma	7,065 (24.1%)	2,482 (27.2%)	*p* < 0.001
Abdominal pain	2,504 (8.5%)	554 (6.1%)	*p* < 0.001
Chest pain	496 (1.7%)	130 (1.4%)	*p* = ns
Seizure	738 (2.5%)	173 (1.9%)	*p* < 0.001
Vomiting	2,427 (8.3%)	308 (3.4%)	*p* < 0.001
Skin rash	1,292 (4.4%)	240 (2.6%)	*p* < 0.001
Diarrhea	675 (2.3%)	119 (1.3%)	*p* < 0.001
Ear pain	946 (3.2%)	153 (1.7%)	*p* < 0.001
Burns	165 (0.6%)	56 (0.6%)	*p* = ns
Eye problems	896 (3.1%)	173 (1.9%)	*p* < 0.001
Others	5,160 (17.6%)	2,255 (24.7%)	*p* < 0.001
Total	29,368 (100%)	9,133 (100%)	

Higher rates of ED visits were registered in March–May 2020 for fever (16.2 vs. 14.3%, *p* < 0.001), respiratory distress (11.1 vs. 9.5%, *p* < 0.001), and traumas (27.2 vs. 24.1%, *p* < 0.001) compared to those of March–May 2019, albeit absolute numbers in the 2020 trimester were much lower. During the 2020 lockdown, lower rates of ED visits were accounted for abdominal pain (6.1 vs. 8.5%, *p* < 0.001), seizure (1.9 vs. 2.5%, *p* < 0.001), vomiting (3.4 vs. 8.3%, *p* < 0.001), skin rashes (2.6 vs. 4.4%, *p* < 0.001), diarrhea (1.3 vs. 2.3%, *p* < 0.001), ear pain (1.7 vs. 3.2%, *p* < 0.001), and eye problems (1.9 vs. 3.1%, *p* < 0.001), compared to March–May 2019.

Burns showed a similar rate over the two considered periods (0.6% in the 2019 trimester and 0.6% in the 2020 one, *p* = ns), but their absolute number was three times before lockdown.

Inpatient hospital admissions following ED consultation were 2,812 in March–May 2019 and 1,455 in March–May 2020, witnessing for a 48.2% reduction during the COVID-19 pandemic. Hospitalization/ED consultation rate was 9.6% in March–May 2019 and 15.9% in March–May 2020 (*p* < 0.001).

Mean hospital stay duration in the 2019 studied trimester was 5.55 (95% CI 5.07–6.03) days and 5.69 (95% CI: 4.91–6.47) days in March–May 2020 (*p* = ns).

As regards the number of frailty/chronic disease patients, there were 1,368 ED consultations for children with special needs in March–May 2019 and 1,282 in March–May 2020 (*p* = ns).

Covid-19 nasal swabs were performed for 385 children; of those, six resulted positive and four of them were hospitalized elsewhere (Pediatric Regional Hub for Infectious Disease); two were quarantined in their house under special medical team follow-up.

## Discussion

During the last two decades, both our EDs have usually hosted a vast majority of non-urgent pediatric consultations which would have been better fit for primary care, and our settings do echo other national and international data (16, 17).

In this study, we aimed to assess the impact of the COVID-19 pandemic and population lockdown on pediatric ED consultations and appropriateness analysis. We saw a significant reduction of non-urgent health care demands during the pandemic, most of all after the lockdown had been announced by the Italian government on March 9, 2020. Several factors might explain this finding. First of all, it should be acknowledged that COVID-19 becomes very rarely a severe clinical condition for children (18). This clinical “mildness” is also witnessed by our cases, and some authors speculated on its immunological age-related factors (19). Furthermore, the initial fear of contracting COVID-19 could have driven less families to EDs for non-urgent consultations. “Stay-at-home” orders also could have resulted in more parental supervision and fewer incidents such as accidents and other injuries. In Italy, specific radio calls and TV advertisements were broadcasted in order to reduce ED direct access without previous primary care assessment/opinion. Lastly, school/nursery closure, combined with seasonal fading of other epidemics such as influenza, gave an extra contribution to the reported reduction. Quite similarly in a French study, pediatric emergency visits revealed that the COVID-19 lockdown and school closure were associated with a significant decrease of pediatric ED visits and subsequent hospital admissions with a significant reduction especially in infectious diseases (20).

Notwithstanding, minor febrile and respiratory diseases in our own experience were more frequently assessed in ED during the COVID-19 epidemic, possibly suggesting parental anxiety about this disease, primary-care reluctance to evaluate these children due to lack of personal protective equipment, and well-established hospital COVID-19 pathways within EDs (21, 22).

However, emergency/resuscitation consultations were significantly higher in March–May 2020 compared to the same trimester in 2019, possibly highlighting both longer time spent home without medical advice or clinical assessment until worsening and family unwillingness to attend either their primary care pediatrician or the ED. In addition, the hospitalization rate among children brought to EDs was significantly higher in March–May 2020 as compared to March–May 2019, thus indicating more severe clinical conditions. These assumptions are in line with a recent pediatric case series underscoring that the risk of delayed access for some emergency conditions can be much higher than that of contracting COVID-19 (23). It is of note that this may be indirectly a pandemic-related drawback which physicians and policy makers should be aware of.

As regards the length of hospital stay for inpatients and consultations for fragile children during the two considered periods, no significant differences were found, thus pinpointing that overall plan for pediatric hospital care and hospital support for children with special needs has passed unchanged through the COVID-19 epidemic.

Emergency medical transports services are often inappropriate, providing pure transportation where no immediate care is actually needed (24). Albeit EMTS involvement was activated more appropriately in March–May 2020 rather than in March–May 2019, even during the COVID-19 pandemic, we observed families misusing the ambulance as mere means of transportation in more than two-thirds of the cases.

Despite a significant reduction of deferrable consultations during March–May 2020, a trend of increased night and weekend visits for non-urgent cases was still present. This definitely questions the current Italian system which fails to provide pediatric primary care 24/7 and force families to seek for medical care directly at the ED even for minor queries.

We are aware of some limitations of our study. The size and characteristics of our work may be not representative of the whole target population especially in places where medical homes as optimal locations for children to receive care for acute, non-emergency, health concerns are available (25). Furthermore, we have no direct proofs of the underlying personal/social motivations which prompted parents to ED consultations. In addition, due to the lack of a regional centralized public health registry, the analysis of our ED databases does not allow having more clues about the final diagnosis of hospitalized patients and comparison with other reports (20). These unanswered questions may need future research.

In conclusion, our work provides a unique snapshot of the pediatric EDs demands in the era of COVID-19. Moreover, current pandemic and lockdown policies unveiled on one hand the still perduring inappropriateness of the majority of pediatric ED consultations. At the same time, thoughtful discussions on current scenario should aim to reinforce the importance of well-structured pediatric primary care, a wise and prompt community filter for disease severity, as well as a strong tackle against ED overcrowding, for the sake of all children and particularly those with special needs.

## Data Availability Statement

The raw data supporting the conclusions of this article will be made available by the authors, without undue reservation.

## Ethics Statement

The studies involving human participants were reviewed and approved by IRB protocol N. 10 approved on 28/04/2020 regional meeting. Written informed consent from the participants' legal guardian/next of kin was not required to participate in this study in accordance with the national legislation and the institutional requirements.

## Author Contributions

FV and LZ conceived the study, drafted the manuscript, and gave final approval of the version to be published. RP, AM, SM, and MB collected and interpreted the data, revised the manuscript critically for important intellectual content, and gave final approval of the version to be published. GB analyzed the data, revised the manuscript critically for important intellectual content, and gave final approval of the version to be published. VT conceived the study, corrected the first draft of the manuscript, and revised the manuscript critically for important intellectual content. PV designed and supervised the study, revised the manuscript critically for important intellectual content, and gave final approval of the version to be published. All authors approved the final manuscript as submitted and agree to be accountable for all aspects of the work.

## Conflict of Interest

The authors declare that the research was conducted in the absence of any commercial or financial relationships that could be construed as a potential conflict of interest.
